# Fighting Tobacco Smoking - a Difficult but Not Impossible Battle

**DOI:** 10.3390/ijerph6010069

**Published:** 2009-01-05

**Authors:** Christopher Man-Kit Leung, Alexander K. C. Leung, Kam-Lun Ellis Hon, Albert Yim-Fai Kong

**Affiliations:** 1 Department of Applied Science, Hong Kong Institute of Vocational Education, 30 Shing Tai Road, Chai Wan, Hong Kong; 2 Department of Pediatrics, The University of Calgary, Alberta Children’s Hospital, #200, 233-16^th^ Ave, N.W., Calgary, Alberta, Canada, T2M 0H5; E-mail: aleung@ucalgary.ca; 3 Department of Pediatrics, Chinese University of Hong Kong, Shatin, Hong Kong; E-mail: ehon@cuhk.edu.hk; 4 Department of Community and Family Medicine, The Chinese University of Hong Kong, Shatin, Hong Kong; E-mail: albertyfkong@hkam.org.hk

**Keywords:** Tobacco, smoking, cigarette, World Health Organization, epidemic

## Abstract

According to the World Health Organization (WHO), tobacco-related disease is the single largest preventable cause of death in the world today, killing around 5.4 million people a year – an average of one person every six seconds. The total number of death caused by tobacco consumption is higher than that of tuberculosis, HIV/AIDS and malaria combined. Unlike other communicable diseases, however, tobacco-related disease has a man-made consensus vector – the tobacco companies that play an active role to promote tobacco consumption, which directly heightens the disease morbidity. Any public health policy designed to curb smoking behavior has to prepare for opposite lobbying actions from tobacco companies that undermine the effects of the health measures. Another unique nature of the tobacco epidemic is that it can be cured, not by medicines or vaccines, but on the concerted actions of government and civil society. Many countries with a history of tobacco control measures indeed experienced a reduction of tobacco consumption. As most of these governments launched a range of measures simultaneously, it is hard to quantify the relative merits of different control strategies that contributed to the drop in the number of smokers. These packages of strategies can come in different forms but with some common features. Political actions with government support, funding, and protection are crucial. Without these, antismoking efforts in any part of the world are unlikely to be successful.

## 1. World Trend of Tobacco-related Diseases

According to the World Health Organization (WHO), tobacco-related disease is the single largest preventable cause of death in the world today. Worldwide, tobacco–related disease accounts for more than five million deaths annually, more than tuberculosis, HIV/AIDS and malaria combined [1]. Although tobacco-related deaths rarely make headlines, tobacco consumption results in the premature death of a third to a half of all people who use it [2], on average 15 years prematurely [3, 4]. By 2030, the death toll will likely exceed eight millions a year. Unless urgent action is taken tobacco could kill one billion people in this century.

There are already numerous publicity campaign and mass education programs on the harmful effects of smoking in many countries all over the world [5, 6]. Although most tobacco users want to quit, yet they are unable to do so because of their dependence on a highly addictive substance. Cigarettes and other tobacco products rapidly deliver the addictive drug nicotine to the brain immediately following inhalation – with an onset time comparable to an intravenously injection [7, 8]. The tobacco industry has referred cigarettes as a “nicotine delivery device” [9].

Until recently, this epidemic of chronic disease and premature death mainly affected rich countries. The trend however is changing – by 2030, more than 80% of tobacco deaths will be in low-income and middle-income countries [10]. In rich countries, smoking is increasingly prevalent among the poor, and is responsible for much of their ill health and premature mortality [11].

In developing countries there has been an upsurge in the number of young men smoking. People in China, for example, smoke about 30% of the world’s cigarettes. This will have catastrophic effects in the 21^st^ century as most other causes of death are likely to continue to decrease and the effects of tobacco to increase. If current smoking patterns persist – that is, if the smoking uptake rate among young adults continues to be substantial and the rate of stopping smoking at older ages continues to be low, by the time the children of today reach middle age smoking will be one of the main causes of premature death in the world [12]. In India, about a quarter of deaths among middle-aged men are caused by smoking. Today, India is the third largest country in the world in both tobacco production and consumption. Of the 1.1 billion smokers worldwide, 182 million live in India [13]. As the number of smokers in the developing world increases with population growth, so will the number of tobacco-related deaths. The shift of the tobacco epidemic to the developing world will lead to unprecedented levels of disease and early death in countries where population growth and the potential for increased tobacco use are highest and where health-care services are least available. Most of the health damages caused by tobacco consumption do not become evident until years or even decades after its use. So, while tobacco use is rising globally, the epidemic of tobacco-related disease and death has just begun.

### 1.1. The Vector - Tobacco Companies

All epidemics have a means of contagion, a vector that spreads disease, and death. For the tobacco epidemic, the vector is not a virus, bacterium or other microorganism – it is an industry and its business strategy. The epidemic of tobacco use and diseases would not exist without the tobacco industry’s marketing and promotion of its deadly products over the past century.

Under the terms of the 1998 Minnesota Consent Judgment and the Master Settlement Agreement (MSA), six leading international tobacco companies were ordered to make millions of pages of their internal documents available to the public as the result of legal action taken by the United States of America. The MSA was originally signed by the four largest tobacco companies, Philip Morris USA, R. J. Reynolds Tobacco Company, Brown & Williamson Tobacco Corp., and Lorillard Tobacco Company and was later joined by over 40 other tobacco companies. 46 U.S. states and 6 U.S. territories signed the agreement. The other U.S. states Florida, Minnesota, Texas and Mississippi had already reached individual agreements with the tobacco industry. With the exception of British American Tobacco and the Liggett Group, the companies were also required to post their documents on public websites and to maintain these sites until June 30, 2010. The estimated 40 million pages in the collections have disclosed, amongst many other insights that the industry has known about health risks associated with tobacco use for years and has contrived to keep this information from the public [14]. These documents indicated that tobacco companies have long targeted youth as “replacement smokers” to take the place of those who quit or die [15–18]. The industry knows that addicting youth is hope for its future. Although anyone who uses tobacco can become addicted to nicotine, people who do not start smoking before age 21 are unlikely to ever begin. The younger the children or adolescents are when they first try smoking, the more likely they are to become regular smokers and the less likely they are to quit [19, 20].

Because of an addicted customer base and high profit margins, tobacco companies have very strong financial positions, and with these, political strengths. Worldwide, the tobacco industry spends tens of billions of dollars a year on marketing. In recent years, global tobacco giants have bought majority stakes in tobacco companies in the Dominican Republics, Indonesia, Mexico and Pakistan, among other countries, to boost sales and use in the developing world [1]. There are also examples of concerted efforts of tobacco giants, mostly from the United Sates, to couple with the influence of their governments to exploit the tobacco markets of other countries. Korea, Taiwan, and Japan have all suffered threats of trade sanctions [21–23].

Governments in developing countries may be under great pressure from the tobacco industry and its political allies, but they have obligation to safeguard the health of their own people. Government leaders hold the cure for the tobacco epidemic. There are many examples of successful actions that can reduce the health hazards of tobacco and each of them outlined below requires the support of the governments.

## 2. Tobacco Control Policies

On August 9, 2000, the U.S. Surgeon General released a report on tobacco, “Reducing Tobacco Use,” which found that smoking rates among teens and adults could be halved within a decade if the nation would fully implement anti-smoking programs using effective approaches that are already available [24]. Reports from the Institute of Medicine [25] and the Centers for Diseases Control and Prevention [26] suggested that comprehensive state tobacco control programs are effective public health investments. The claims are further supported by a recent research in which researchers were able to quantify the link between comprehensive tobacco control programs and a decrease in adult smoking – observing a decline in prevalence from 29.5 percent in 1985 to 18.6 percent in 2003 [27]. The study, “The Impact of Tobacco Control Programs on Adult Smoking”, published in 2008, is the first of its kind to use multi-state survey data on smoking to examine the association between cumulative state tobacco control program spending and changes in adult smoking prevalence [27]. Combining educational, clinical, regulatory, economic, and social strategies, these comprehensive programs encompass coordinated efforts to establish smoke-free policies and social norms, to promote and assist tobacco users to quit, and to prevent initiation of tobacco use.

The U.K. Government is also reporting similar success in the range of policies it has successfully implemented to drive down smoking prevalence since the publication of the seminal white paper, *Smoking Kills*, in 1998 [28]. Indeed it has been reported that smoking rates in England have fallen from 28 per cent in 1998 to 22 per cent in 2006 with a reduction in smoking amongst routine and manual workers from 33 to 28 percentage points, with a similar picture across the U.K. [29].

In general, most of the countries around the world strongly favor policies that might prevent tobacco use among young people. These policies might include mandated tobacco education in schools, a complete ban on smoking on school grounds, further restrictions on tobacco advertising and promotional activities, stronger prohibitions on the sale of tobacco products to minors, and increases in earmarked taxes on tobacco products. Interventions to prevent initiation among young people, as well as actions that involve restrictions on adult smoking or increased taxes, have received strong support among smoking and nonsmoking adults.

One key element in determining the relative merits of the various control strategies is the availability of data. Comprehensive data are important for convincing the leaders of governments, the general public and other stakeholders how the tobacco consumption harms the countries and what policy should be implemented and concentrated in order to protect their citizens. The data can also enable policy makers to gain “ownership” of the chosen interventions by providing them with the research findings on which the interventions are based. There might be other control policies that work on a local scale but the good experience cannot be shared without systematic data analysis and monitoring. The following discussion of effective tobacco control policies is based on well-documented evidence.

### 2.1. The Framework Convention on Tobacco Control (FCTC)

On May 21, 2003, the World Health Assembly (WHA), the governing body of the WHO, unanimously adopted resolution WHA 56.1, which included adoption of the WHO Framework Convention on Tobacco Control (FCTC) [30]. The WHO FCTC consists of a series of negotiated protocols within a general framework. The first three protocols are negotiations covering smuggling, advertising, and treatment of tobacco addiction. Countries agreeing to the negotiated protocols are to adopt appropriate legislation and, if necessary, implement the appropriate measures.

The WHO FCTC opened for signature from 16 June to 22 June 2003 in Geneva, and thereafter at the United Nations Headquarters in New York, the Depositary of the treaty, from 30 June 2003 to 29 June 2004. The treaty, which is now closed for signature, has 168 signatories, including the European Community, which makes it the most widely embraced treaty in the UN history. Member states that have signed the Convention indicate that they will strive in good faith to ratify, accept, or approve it, and show political commitment not to undermine the objectives set out in it. Countries wishing to become a Party, but that did not sign the Convention by 29 June 2004, may do so by means of accession, which is a one-step process equivalent to ratification.

The WHO FCTC is the first treaty negotiated under the auspices of the WHO. It is an evidence-based treaty that represents a paradigm shift in developing a regulatory strategy to address addictive substances; in contrast to previous drug control treaties, the WHO FCTC asserts the importance of demand reduction strategies as well as supply issues.

On the demand reduction strategies, the WHO FCTC includes price and tax measures to reduce the demand for tobacco, and non-price measures to reduce the demand for tobacco, namely: protection from exposure to tobacco smoke; regulation of the contents of tobacco products; regulation of tobacco product disclosures; packaging and labeling of tobacco products; education, communication, training and public awareness; tobacco advertising, promotion and sponsorship; and, demand reduction measures concerning tobacco dependence and cessation. On the supply issue, the treaty addresses: illicit trade in tobacco products; sales to and by minors; and, provision of support for economically viable alternative activities [31].

Between 1999 and 2001, British American Tobacco, Philip Morris, and Japan Tobacco International executed Project Cerberus to develop a global voluntary regulatory regime as an alternative to the FCTC. They aimed to develop a global voluntary regulatory code to be overseen by an independent audit body and to focus attention on youth smoking prevention. Although the companies did not stop the FCTC, they continue to promote the International Tobacco Products Marketing Standards youth smoking prevention as an alternative to the FCTC [32]. Fortunately many public health civil society groups helped policymakers and governments to understand the situation and many countries signed on the FCTC disregarding the alternative promoted by the tobacco companies.

The major impact of the FCTC is to define international rules for tobacco control. Many countries then aim to follow the Convention to guarantee the adoption of some, if not all, measures that, based on the opinions of WHO, will effectively reduce tobacco consumption. There are some important measures that tended to be adopted more by many member states, probably due to their apparent effectiveness and their approaches are more acceptable and applicable to different countries. These approaches are outlined as follows.

### 2.2. Smoke-Free Policies

Smoke-free policies, one of the most important measures to protect people from tobacco smoker, refer to restriction of smoking in public places. Smoke-free policies include legislative and other measures to protect against harmful exposure to second-hand smoke and are an integral part of the WHO FCTC. There are various degrees of restrictions and the only country to have banned the sale and smoking of tobacco in all public places is Bhutan, in early 2004.

Environmental tobacco smoke or “second-hand smoke” is the complex mixture of chemicals generated during the burning of tobacco products. The primary rationale of government smoke-free laws is to protect people from the alleged harmful effects of second-hand smoke, which include an increased risk of heart disease, cancer, emphysema, and other diseases [33, 34]. Laws implementing bans on indoor smoking have been introduced by many countries in various forms over the years, with some legislators citing scientific evidence that shows tobacco smoking is harmful to the smokers themselves and to those inhaling second-hand smoke.

Other than the immediate health benefits such as reduction of respiratory symptoms in workers in smoke-free workplace, the long-term health benefits of smoke-free laws on fatal diseases such as cancer and heart attacks are less easy to demonstrate. Conclusive research on such areas need enormous time and resources investment, not to mention the technical difficulties involved.

There is an overwhelming scientific consensus that exposure to second-hand smoke cause disease, disability and death. Second-hand smoke contains over 4,000 chemicals and 69 known carcinogens including formaldehyde, cyanide, arsenic, carbon monoxide, methane, benzene, and radioactive polonium 210. Recent report from the US Surgeon General collated and collected vast amount of information over the years among various countries and finally concluded that second-hand smoke exposure increases the risk of coronary heart disease by 25–30% and the risk of lung cancer in non-smokers by 20–30% [33]. Since second-hand smoke contributes to all these health problems, the implementation of smoke-free laws would result in a reduction in the risk of cancer, respiratory disease, heart disease and stroke [35].

From a public health standpoint, the greatest impact of smoke-free laws is to curb the smoking habit. The WHO considers smoke-free laws to have an influence to reduce demand for tobacco by creating an environment where smoking becomes increasing more difficult and to help shift social norms away from the acceptance of smoking in everyday life. Smoke-free environments also help smokers who want to quit. Smoke-free laws in workplaces were found to cut absolute smoking prevalence by 4% [36]. Smoke-free policies in workplaces in several industrialized nations have reduced total tobacco consumption among workers by an average of 29% [36]. Along with tax measures, cessation measures, and education, smoking ban policy is currently viewed as an important element in lowering smoking rates and promoting public health [1].

It is generally agreed that comprehensive smoke-free regulations can reduce cigarette consumption, but that such policies work best when there is a strong social consensus against smoking in public places and therefore self-enforcement of the restrictions.

### 2.3. Health Publicity

There are many studies on the effects of health publicity programs in curbing tobacco consumption and these effects are dependent on the medium used and the recipients’ social background. For example, studies of rates of stopping smoking between 1950 and 1980 in the United States suggest that health scares in the 1950s, which were largely carried by the print media, had relatively little influence on the prevalence of smoking in more deprived groups relative to the population as a whole. These groups were much more responsive, however, to later publicity in the electronic media –especially the “fairness doctrine” antismoking campaign on television between 1967 and 1970 [37]. Townsend and colleagues’ study found that health publicity had relatively less influence on the smoking habits of more disadvantaged socioeconomic groups, and is therefore a source adding inequality [38]. Smokers in all social classes however responded equally to Sydney’s “quit for life” campaign on television in 1983 [39]. It appears that television, which is commonly watched by members across different social classes, is a potentially class free medium for health promotion in comparison with the print media, though the latter probably have a correspondingly greater influence on decision makers [40].

Reviews of the impact of specific types of information (such as “information shocks” – the publication of new evidence on the health consequences of smoking – and warnings on cigarette packs) indicate that these can effectively reduce the prevalence of smoking in a population [41].

The evidence suggests that government policies to increase consumer information about the health consequences of smoking can form part of an effective tobacco control program. Health publicity program designed for adults, however, has less conclusive beneficial effects on preventing young people to smoke. The Royal College of Physicians on the other hand argued in its 1992 report that interventions oriented to children were unlikely to be effective while the prevalence of adult smoking remains high, and so there is a need to help more adult smokers to quit [42]. Other benefits are likely to arise from a concentration on reducing smoking among adults with children. Reduced smoking by parents is likely to directly improve the health of non-smoking children and may reduce the extra healthcare costs among these children [43].

Interventions to prevent tobacco use initiation and encourage cessation among young people need to reshape the environment so that it supports tobacco-free norms. As early adolescence (age 11–15 years) is the period when young people are most likely to try smoking for the first time, intervening during adolescence is critical [24]. Since the late 1980s, most governments’ strategies to prevent the onset of cigarette smoking were often based on the premise that adolescents who engaged in smoking behavior had failed to comprehend the health hazards of smoking. The assumption was that these young people had a deficit of information that could be addressed by presenting them with health messages in a manner that caught their attention and provided them with sufficient justification not to smoke. Improvements in knowledge levels, or cognitive factors, would thus lead directly to changes in behavior. Unfortunately, many of the existing health education messages, delivered in the conventional fashion, appear to be overpowered by the glamour and image of smoking, vividly portrayed by the powerful tobacco companies [44]. One of the possible reasons is that young people are in their prime time of health and do not realise the full impact of the damaging effects of smoking as many of its related diseases tend to take sometime to develop. If young people are asked to make a “Choice” based on all the medical facts and data, they may take a risk to smoke as they might underestimate the potential risks of cigarettes. The dilemma therefore develops: should the health authorities ask the children to make a choice simply by giving them all the medical facts and passively trusting them to make a healthy choice, or alternatively impose them upon a smoke-free lifestyle with the danger of indirectly inducing them to experiment with smoking as a counter reaction to suppression.

Health publicity as a standalone approach in itself is therefore not good enough. Community programs and school-based policies and interventions should be part of a comprehensive effort, implemented in coordination across the community and school environments and in conjunction with increasing the unit price of tobacco products, sustaining anti-tobacco media campaigns, making environments smoke-free, and engaging in other efforts to create tobacco-free social norms [45].

On the other hand, tobacco companies are also taking the “Choice” battle by sponsoring various forms of “Choice” programs: disguised as tobacco prevention program under the name of social responsibility. These modified “Choice” programs use provocative messages for health advice in the context that the children is not old enough to make their own choice so they are “advised” not to smoke as they are too young. These types of modified choice programs make use of the rebellious nature of young people and arouse their desire to smoke, to be free to do something that challenges the authorities [46, 47]. It is probably one of these factors that renders health publicity programs for teenagers less conclusive due to the mixing up of tobacco prevention programs with “disguised” tobacco advocating programs.

### 2.4. Tobacco Taxation

When the price of cigarettes goes up, demand drops substantially. Increasing cigarette excise taxes is one way to cause the price of cigarettes to go up. Most researches suggest that cigarette companies pass the full amount of the tax increases through to consumers in the form of higher prices [48]. There have been many studies measuring the relationship between prices and the consumption of cigarettes, and most have similar results [45, 48–50]. In the short run, most studies find that a 25 percent increase in the price of cigarettes due to a tax increase will reduce consumption of cigarettes by just over 11 percent [49]. There are also numerous studies all over the world clearly demonstrating that increases of tobacco tax would decrease the number of teenage smoking; younger smokers are more likely than older smokers to quit smoking in response to a price increase [50]. It would probably reduce the attractiveness of smoking among young people, as they have to depend on the financial support of their parents, and this will restrict their choice of expenditure items. In fact, the evidence was so overwhelming that the WHO claimed that “Increasing the price of tobacco through higher taxes is the single most effective way to decrease consumption and encourage tobacco users to quit [51]”.

Tobacco price elasticity is estimated by the World Bank to be around –0.4 for developed countries, which means that a 10% rise in price leads to a 4% decline in consumption, but the reduction effects on consumption tended to be higher in developing than developed countries, and also higher among lower-income smokers, and younger smokers [52]. The increase in retail price of cigarettes will decrease their purchasing power to buy cigarettes, hence their daily consumption. Eventually, it will benefit their health and prevent the tobacco-related diseases. In general, price increases are found to be the most cost-effective antismoking intervention. In fact, tobacco company’s internal documents provide clear evidence on the impact of cigarette prices on cigarette smoking, describing how tax and other price increases lead to significant reductions in smoking, particularly among young persons [53].

### 2.5. Ban on Tobacco Promotion

Cigarette manufacturers are in business to make a profit which depends on their ability to recruit new smokers. There is therefore a strong incentive for a tobacco company to compete for market share among beginning smokers, since the long-term prosperity of a given cigarette brand and company will depend on the percentage of new smokers that can be captured. A misguided debate has arisen about whether tobacco promotion “causes” young people to be nicotine-addicted because single-source causation is probably too simple an explanation for any social outcome. The more important issue is what effect tobacco promotion might have. The public health literature clearly demonstrates that youth are exposed to a wide variety of industry marketing efforts, and that there is a consequent adverse effect upon adolescent smoking initiation rates [47, 52–54]. Current research suggests that pervasive tobacco promotion has two major effects: it creates the perception that more people smoke than they actually do, and it provides a conduit, between actual self-image and ideal self-image. In other words, smoking is made to look cool [37].

It has been suggested that the reactions of the tobacco industry to control strategies serve as a litmus test of potential effectiveness [55]. Banning promotion, setting prices policy, and establishing smoke-free areas are the three strategies most vociferously opposed by the tobacco industry, a good indication of their importance [56]. These three strategies are therefore highlighted as the top three effective tobacco control approaches as listed in this paper. The remaining strategies described are not meant to be of less importance. Yet there are limitations, mainly streamed from resources implication and other political constraints that restrict direct application of these strategies in many countries.

### 2.6. Product Litigation

The U.S. tobacco giants are usually the targets for legal actions as they are the dominating exporters of tobacco products to the international market. Besides there are still many countries (over 40) with a tobacco monopoly, for example China, making it more difficult for citizens to sue, as it would in reality mean suing their own governments. Historically, tobacco litigation began with a personal injury lawsuit in the USA in 1954. For over 40 years, the tobacco industry boasted it had never lost a single case, but this was changed. One case in Minnesota that began in 1994 ruled that millions of pages of internal tobacco industry documents be put into the public domain [57]. These showed that the industry has concealed information on the true harmfulness of smoking and misled governments, the media and their customers. The disclosed documents also facilitate further lawsuits as internal documents from the tobacco companies turned out to be important evidence.

When a Los Angeles jury assessed $28 billion in punitive damages against Philip Morris in Bullock *versus* Philip Morris Companies the tobacco control community cheered [58]. The jury had calculated that only one in 28,000 Californians who have suffered from tobacco caused disease ever sues, so to make Philip Morris confront the real cost of its misbehavior, they multiplied a typical $1 million compensatory damage award (for medical bills, lost wages, and pain and suffering) by 28,000. In theory, further success of similar cases would bring down the tobacco giants financially.

However not many plaintiffs, even in the form of a class action, can put up the energy and resources to battle with the tobacco companies on the endless litigation process – the progress of tobacco litigation can take years with no guarantee of successful endings. It is quite certain that tobacco companies will exhaust any appeal procedures to revert any decision that holds the tobacco companies responsible for the damages that their products caused to the victims. A major legal victory that pushes the tobacco companies into financial crisis remains to be seen.

### 2.7. Quit Services

The addictive nature of tobacco use can be broadly divided into two components - physical and psychological dependence [7]. Nevertheless, the role of how these two components interact with each other is not well defined. It is also not clearly understood why some smokers are easier than others to quit. On the other hand, there is overwhelming evidence that quitting provides enormous health benefits: Interventions that increase quitting can decrease premature mortality, morbidity, and tobacco-related health care costs [59, 60]. Quitting by age 30 eliminates nearly all excess risk associated with smoking, and smokers who quit smoking before age 50 cut in half their risk of dying in the next 15 years [61, 62]. Although there are many ex-smokers who quit successfully on their own, many more tobacco users want to quit but are unable to do so. More than 40% of smokers in United States try to quit each year. But without assistance, fewer than 10% succeed [63].

To increase the success rate of tobacco use cessation, there are many quitting methods ranging from brief advice by medical providers to quit smoking, more intensive interventions (individual, group, or telephone counseling) that provide social support, and coaching on problem-solving skills, to pharmacotherapy such as nicotine replacement therapies, bupropion and varenicline. As a whole, nicotine replacement therapies and other pharmacological quitting aids can approximately double the chances that an individual will succeed in quitting compared with unaided attempts [60].

Unfortunately the vast majority of countries do not help tobacco users who want to quit. Currently only 5% of the world’s population have access to treatment for tobacco dependence [1]. Due to the high relapse rate, tobacco dependence might be better viewed as a chronic disorder, requiring repeated episodes of treatment [64]. While some types of cessation treatment are less expensive than others, all require some kind of expenditure which can be difficult for some countries to fund.

## 3. Conclusions

It appears that countries which have not yet taken vigorous action against tobacco smoking have the highest percentage of smokers among their adult population. With rising cigarette consumption in these countries, one can expect substantial increases in lung cancer and their smoking-related diseases in the next 30–40 years. These countries would need to decide carefully on which tobacco control strategies they should channel their limited financial and time resources.

On a macroscopic scale, it is obvious that increase of tobacco tax would be the top priority tobacco strategy to be adopted by the government. It is also possible to generate revenue through this taxation to fund other “non-price’ measures for tobacco control. A set of “non-price” measures has also been discussed, including comprehensive bans on advertising and promotion, information campaign, clean-air restrictions, cessation treatment, and legal litigation.

All of these effective tobacco policies have one feature in common: political actions with government support, funding, and protection are crucial. Without these, antismoking efforts in any part of the world are unlikely to be successful. For countries that have limited resources, they would need to concentrate on the most important policies among them. The answer as provided by Judith Mackay, around twenty years ago, is to place banning promotion, setting price policy and establishing smoke-free areas as the top three priorities [56]. Her wisdom remains valid in the 21^st^ century.
